# Characteristics of the Gut Microbiome and Its Relationship With Peripheral CD4^+^ T Cell Subpopulations and Cytokines in Rheumatoid Arthritis

**DOI:** 10.3389/fmicb.2022.799602

**Published:** 2022-02-03

**Authors:** Qi Wang, Sheng-Xiao Zhang, Min-Jing Chang, Jun Qiao, Cai-Hong Wang, Xiao-Feng Li, Qi Yu, Pei-Feng He

**Affiliations:** ^1^School of Basic Medical Sciences, Shanxi Medical University, Taiyuan, China; ^2^Key Laboratory of Cellular Physiology at Shanxi Medical University, Ministry of Education, Taiyuan, China; ^3^Shanxi Key Laboratory of Big Data for Clinical Decision Research, Taiyuan, China; ^4^Department of Rheumatology and Immunology, The Second Hospital of Shanxi Medical University, Taiyuan, China; ^5^School of Management, Shanxi Medical University, Taiyuan, China

**Keywords:** rheumatoid arthritis, gut microbiota, cytokines, CD4, T cells, immune system diseases, DAS28

## Abstract

This study investigated the association between intestinal microbiota abundance and diversity and cluster of differentiation (CD)4^+^ T cell subpopulations, cytokine levels, and disease activity in rheumatoid arthritis RA. A total of 108 rheumatoid arthritis (RA) patients and 99 healthy control (HC) subjects were recruited. PICRUSt2 was used for functional metagenomic predictions. Absolute counts of peripheral CD4^+^ T cell subpopulations and cytokine levels were detected by flow cytometry and with a cytokine bead array, respectively. Correlations were analyzed with the Spearman rank correlation test. The results showed that the diversity of intestinal microbiota was decreased in RA patients compared to HCs. At the phylum level, the abundance of Firmicutes, Fusobacteriota, and Bacteroidota was decreased while that of Actinobacteria and Proteobacteria was increased and at the genus level, the abundance of *Faecalibacterium*, *Blautia*, and *Escherichia-Shigella* was increased while that of *Bacteroides* and *Coprococcus* was decreased in RA patients compared to HC subjects. The linear discriminant analysis effect size indicated that *Bifidobacterium* was the most significant genus in RA. The most highly enriched Kyoto Encyclopedia of Genes and Genomes pathway in RA patients was amino acid metabolism. The relative abundance of *Megamonas*, *Monoglobus*, and *Prevotella* was positively correlated with CD4^+^ T cell counts and cytokine levels; and the relative numbers of regulatory T cells (Tregs) and T helper (Th17)/Treg ratio were negatively correlated with disease activity in RA. These results suggest that dysbiosis of certain bacterial lineages and alterations in gut microbiota metabolism lead to changes in the host immune profile that contribute to RA pathogenesis.

## Introduction

Rheumatoid arthritis (RA) is a chronic autoimmune disease characterized by irreversible peripheral joint damage (England et al., 2018). The etiopathogenic mechanism of RA involves abnormal immune activation with various autoantibodies, an imbalance of lymphocyte subpopulations, and cytokine dysregulation.

Alterations in the gut microbiome profile are associated with immune dysfunction in several rheumatic diseases (Li and Wang, 2021). The abundance of intestinal microbiota is known to differ between RA patients and healthy subjects (Vaahtovuo et al., 2008; Scher et al., 2013; Taneja, 2014). Moreover, gut dysbiosis may negatively affect immune function in RA (Li and Wang, 2021). Interaction between gut microbiota and the host is important for maintaining immune homeostasis (Chu and Mazmanian, 2013). Gut bacteria have been shown to influence the polarization of lymphocyte subpopulations and their cytokines and thereby regulate immune functions (Abdulla et al., 2021).

Dysbiosis in the gut microbiome is associated with various autoimmune diseases, and the detailed mechanisms of how changes in the abundance and diversity of these microorganisms contribute to disease pathogenesis in the host is unknown (Li et al., 2017). To address this question, in this study we investigated the associations between gut microbiota community composition and metabolic pathways, cluster of differentiation (CD)4^+^ T cell subpopulations, cytokine levels, and disease activity in RA.

## Materials and Methods

### Study Population

A total of 108 RA patients (73 females, 35 males) were recruited at the Department of Rheumatology, Second Hospital of Shanxi Medical University (Zhao et al., 2019) between December 2018 and August 2019. No medication was provided to patients, and the RA group specimens were collected on the day of hospitalization. The median age was 52.7 years and the age range was 21–70 years. We also recruited 99 healthy controls (HCs) (68 females, 31 males) from our health examination center (Li Y. et al., 2021). The mean age of healthy volunteers was 56.56 years. There were no statistically significant differences (*t*-test) in the composition of the 2 groups with regard to age, and sex. All patients met the 2010 American College of Rheumatology/European League Against Rheumatism classification criteria for RA (Lee and Kim, 2017). Exclusion criteria were subjects who had another autoimmune-related or gastrointestinal tract or inflammatory bowel disease, take any probiotics or antibiotics within 1 month, serious infection, or a malignant tumor within the previous 6 months (Zhang et al., 2021). Each participant signed the consent form. This study was approved by the institutional ethics committee of the Second Affiliated Hospital of Shanxi Medical University (approval no. 2019-YX-107).

We recorded all clinical symptoms and laboratory measures of disease activity including C-reactive protein, erythrocyte sedimentation rate, swollen joint count, and tender joint count; these were used to calculate the disease activity score (DAS28) (Li et al., 2020).

### Fecal Sample Collection and 16S rRNA Gene Sequencing

Fresh fecal samples were collected and immediately stored at −80°C in a sterile box for microbial DNA extraction (Li Y. et al., 2021). After obtaining consent, peripheral blood samples were obtained from 106 patients and 109 patients were tested for cytokine levels. Microbial DNA was extracted from about 250 mg of fecal sample using the QIAamp PowerFecal DNA Kit (Qiagen, Valencia, CA, United States) according to the manufacturer’s instructions. It was then quantified by agarose gel electrophoresis on a NanoDrop One spectrophotometer (Thermo Fisher Scientific, Waltham, MA, United States) (Ahmad et al., 2019; Li Y. et al., 2021). The primers 341F (CCTACGGGNGGCWGCAG) and 805 R (GACTACHVGGGTATCTAATCC) were used to PCR amplify the V3–V4 region of the 16S rRNA gene (Liang et al., 2017) using KAPA HiFi HotStart Ready Mix (Roche, Indianapolis, IN, United States). The resultant amplicons were sequenced on a MiSeq platform (Illumina, San Diego, CA, United States).

QIIME2 was used to process representative sequence clusters with a similarity cutoff of 100% (Ahmad et al., 2019). Operational taxonomic units (OTUs) were partitioned into taxonomic lineages in the SILVA 16S rDNA database (Frisbee et al., 2019). Based on the rarefied OTUs, α and β diversity were calculated using the R package “PhyloSeq.” Shannon (also known as Shannon-Weaver index) (Ott et al., 2014; Gabriel et al., 2019), simpson, and invSimpson index (richness and evenness) and richness (observed OTUs, chao1, ACE) (Ramadan et al., 2021) was used to measure alpha-diversity. Biomarker species were identified based on linear discriminant analysis effect size (LefSe).

### Metagenome Functional Predictions

Microbial functions were predicted using PICRUSt2 (Chen et al., 2020), which contains reference genomes and gene families and provides interoperability with OTU picking and a denoising algorithm that incorporates the Benjamini–Hochberg correction (Muraoka et al., 2020), allowing assignment of OTUs to reference genomes and linking of taxonomic information to Kyoto Encyclopedia of Genes and Genomes (KEGG) annotations (Douglas et al., 2020). PICRUSt2 was used to assess the potential metabolic functions of the gut microbiome in RA (Suárez-Moo et al., 2020).

### Flow Cytometry

CD4^+^ T cells in whole blood collected in a 3-ml anticoagulant tube were detected using a FACSCalibur flow cytometer (BD Biosciences, San Jose, CA, United States) (Kojima et al., 1976; Niu et al., 2020). Four multicolor monoclonal antibodies were used for immunofluorescence labeling of circulating CD4^+^ T cell subpopulations. An 80-μl volume of blood was combined with 10 μl ionomycin, 10 μl phorbol 12-myristate 13-acetate, and 1 μl GolgiStop and incubated at 37°C for 5 h (Niu et al., 2020). Fluorescein isothiocyanate-conjugated anti-CD4 antibody was added to the tube, followed by incubation for 30 min in the dark at room temperature (20–25°C). The cells were immobilized using Cytofix/Cytoperm reagent and incubated at 4°C for 30 min; they were then labeled with allophycocyanin (APC)-conjugated anti-interferon (IFN)-γ, phycoerythrin (PE)-conjugated anti-interleukin (IL)-17A, and PE-conjugated anti-IL-4 antibodies to detect Th1, Th17, and Th2 cells, respectively, followed by incubation for 30 min in the dark at room temperature (20–25°C). To detect regulatory T cells (Tregs), 80 μl of anticoagulant-treated blood was labeled with APC-conjugated anti-CD25 and PE-conjugated anti-forkhead box (Fox) P3 antibodies. After washing, the samples were analyzed by flow cytometry. The percentages and absolute counts of CD4^+^ T cell subpopulations were determined using BD Multitest software (BD Biosciences) (Niu et al., 2021).

A flow cytometric bead array (Jiangsu, China) was used to analyze the levels of IL-2, IL-4, IL-6, IL-10, IL-17, tumor necrosis factor (TNF)-α, and IFN-γ, followed by flow cytometry analysis (Chen et al., 2012). The standard curve of each cytokine was in the range of 1–5,000 pg/ml.

### Statistical Analysis

Alpha diversity was compared between groups with Welch’s *t*-test. Correlations were evaluated with the Spearman rank correlation test (Anuradha et al., 2017). Differences with *P* < 0.05 were considered statistically significant.

## Results

### Clinical Characteristics of the Study Population

A total of 207 stool samples were collected. The detailed clinical characteristics of the study population are shown in Table 1.

**TABLE 1:** Clinical characteristics of the study population.

	RA (*n* = 108)	HC (*n* = 99)
Age, years, mean (median)	52.56 (53)	50.44 (50)
Female	67.6%	68.7%
**Disease activity parameters**		
ESR, mm/h, mean (median)	56.55 (43), 3–124	
CRP, mg/l, mean (median)	33.01 (13.75), 0–325	
TJC-28, mean (median)	10.09 (6.5), 0–28	
SJC-28, mean (median)	0.19 (0), 0–2	
DAS28, mean (median)	4.51 (4.45), 1.9–7.1	
**Lymphocyte subpopulations**		
Th1, mean (median)	162.51 (131.38), 11.25–549.26	
Th2, mean (median)	7.14 (6.23), 3.12–19.06	
Th17, mean (median)	8.84 (6.76), 1.69–19.22	
Tregs, mean (median)	31.58 (28.19), 7.93–87.75	
**Cytokines**		
IL-2, mean (median)	4.67 (3.03), 0–30.55	
IL-4, mean (median)	5.53 (2.805), 0–82.48	
IL-6, mean (median)	28.00 (15.77), 0–93.63	
IL-10, mean (median)	7.01 (5.62), 0–30.2	
IL-17, mean (median)	18.20 (9.575), 0–57.16	
TNF-α, mean (median)	6.60 (5.62), 0–27.02	
INF-γ, mean (median)	10.39 (5.535), 0–77.83	

### Disease Duration Is Associated With Decreased Microbial Diversity

**FIGURE 1:**
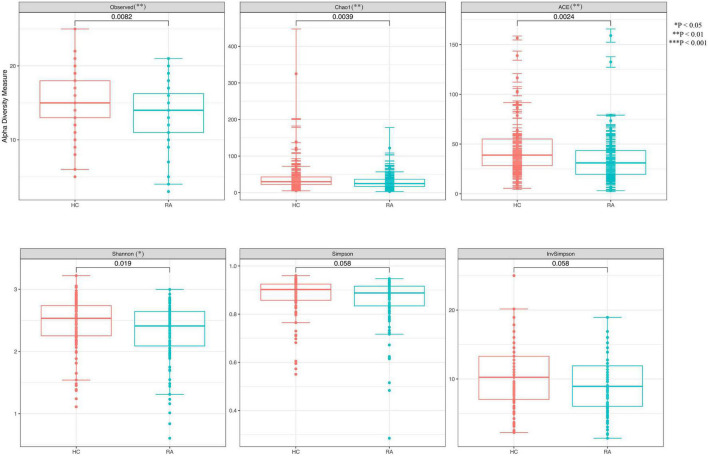
Differences in α diversity of the gut microbiome between RA patients and HCs. **P < 0.01, ***P < 0.001.

**FIGURE 2:**
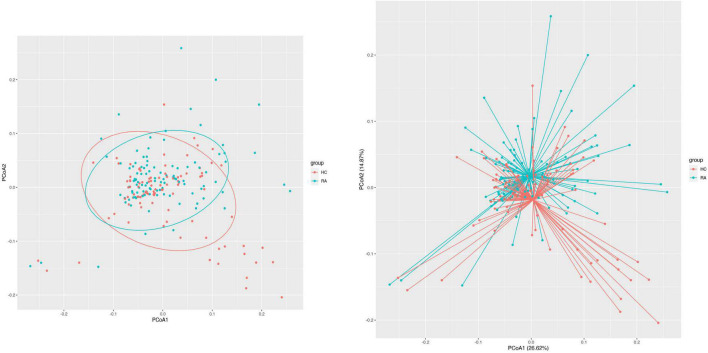
β Diversity of the gut microbiome in RA patients and HCs. Principal coordinate analysis plot generated from the weighted UniFrac analyze. The x and y axes represent the first and second coordinates, respectively; and values in parentheses show the percentage of the community variation explained by each coordinate. Blue and red symbols represent bacteria in the RA and control groups, respectively.

**FIGURE 3:**
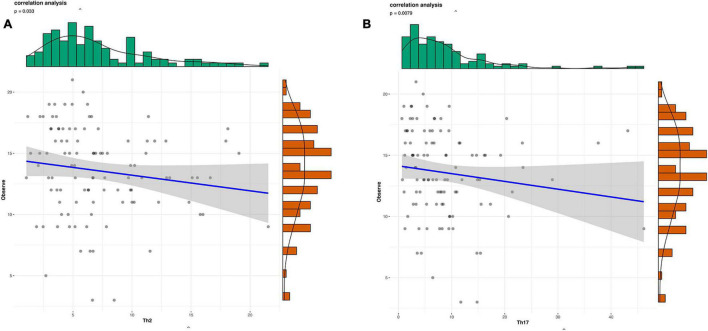
Scatterplots of the correlation between cytokine levels and α diversity. **(A,B)** Correlation between Th1 **(A)** and Th17 **(B)** and Observed value (richness).

We obtained 13,147 high-quality OTUs including 12 phyla, 19 classes, 42 orders, 72 families, and 189 genera. The Observed, ACE, Chao1 (richness) and Shannon (richness and evenness) indices showed difference in RA patients compared to HCs (*P* < 0.05) (Figure 1), although there was no difference in Simpson index between the 2 groups (Figure 1). Above all, gut microbiota was markedly less diverse in composition in RA patients than in the HC group. These indexs lay a foundation for species analyzed. The weighted-UniFrac distance principal coordinate analysis based on OTUs showed that the gut microbial community structure between RA and HC groups differed (Figure 2). Helper T cell (Th)2 (Figure 3A) and Th17 (Figure 3B) counts were negatively correlated with α diversity in RA patients (*P* < 0.05 and < 0.01, respectively).

**FIGURE 4:**
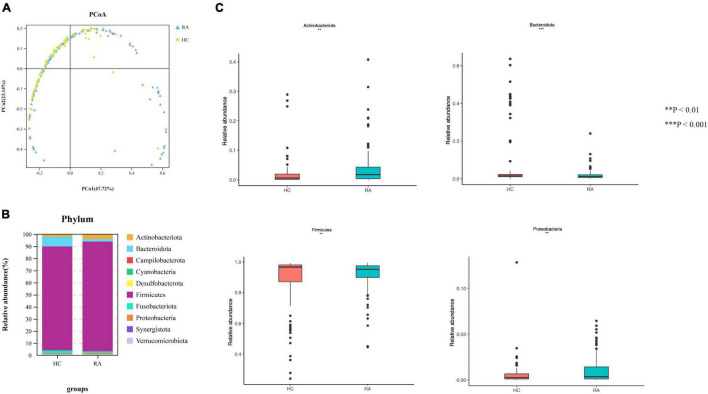
Microbiota community composition of RA patients at the phylum level. **(A)** Principal coordinate analysis plots at the phylum level for RA patients and HCs. The x and y axes represent the first and second coordinates, respectively, and values in parentheses show the percentages of the community variation explained by each coordinate. Blue and green symbols represent bacterial phyla in the RA and control groups, respectively. **(B)** Relative abundance of phyla in all subjects and in each group. **(C)** Phylum-level comparison of the relative abundance of gut microbiota between RA patients and HCs. *P < 0.05, **P < 0.01, ****P < 0.001.

**FIGURE 5:**
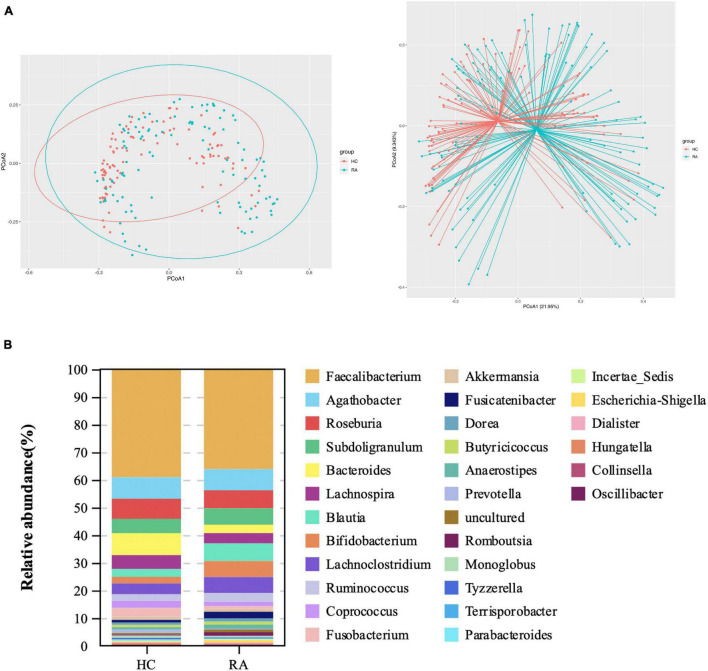
Microbiota community composition of RA patients at the genus level. **(A)** Principal coordinate analysis plots generated from the Bray–Curtis analysis. The x and y axes represent the first and second coordinates, respectively, and values in parentheses show the percentages of the community variation explained by each coordinate. Blue and red symbols represent bacterial genera in the RA and control groups, respectively. **(B)** Relative abundance of genera in all subjects and in each group.

**FIGURE 6:**
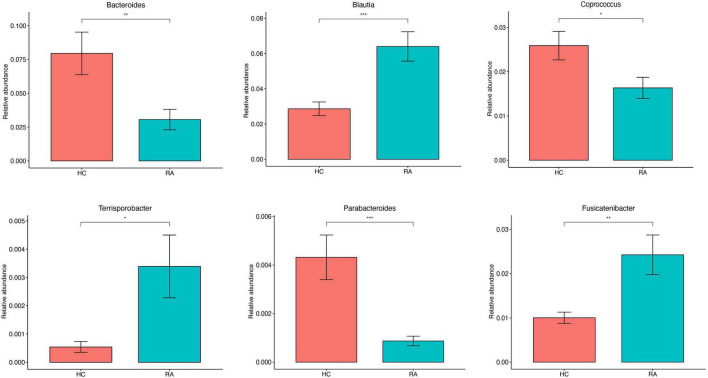
Genus-level comparison of the relative abundance of gut microbiota between RA patients and HCs. *P < 0.05, **P < 0.01, ***P < 0.001.

Gut microbial community structure differed between RA and HC groups, as revealed by phylum and genus Bray–Curtis distances (Figures 4A, 5A). At the phylum level, the relative abundance of *Firmicutes*, *Fusobacteriota*, and *Bacteroidota* was decreased (*P* < 0.05) whereas that of *Actinobacteria* and *Proteobacteria* was increased (*P* < 0.05) in RA patients compared to HCs (Figures 4B,C). At the genus level, the abundance of *Faecalibacterium*, *Blautia*, *Terrisporobacter*, *Escherichia-Shigella*, and *Fusicatenibacter* was increased while that of *Bacteroides*, *Coprococcus*, and *Parabacteroides* was decreased (*P* < 0.05) in RA patients compared to HCs (Figures 5B, 6).

### Gut Flora Is Altered in Rheumatoid Arthritis

**FIGURE 7:**
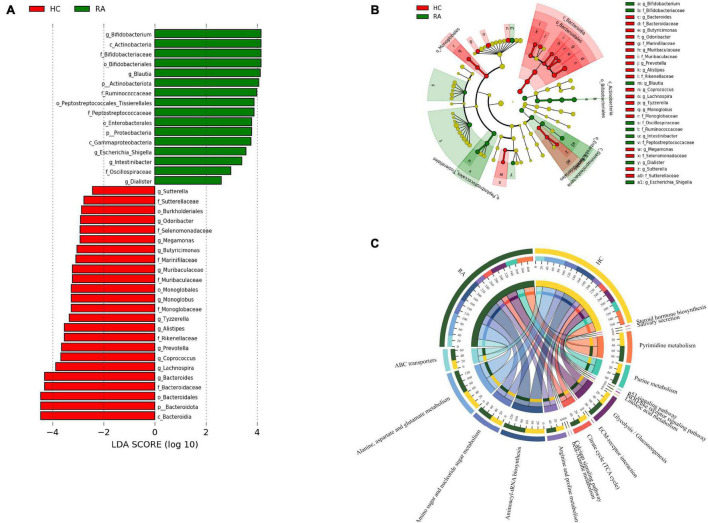
Patients with RA characterized by expansion of rare microbial lineages. **(A,B)** LEfSe analysis was performed to identify differentially abundant taxa, which are highlighted on the phylogenetic tree in cladogram format **(A)** and for which the linear discriminant analysis scores are shown **(B)**. Red and green colors indicate an increase or decrease in taxa, respectively, in RA patients compared to HCs. **(C)** KEGG pathways significantly enriched in RA patients compared to HCs.

A total of 28 differentially abundant taxa were observed by LEfSe analysis (Mollazadeh et al., 2019). The abundance of phylum *Actinobacteria*, including the genus *Bifidobacterium*, was increased in RA patients compared to HCs. The genus *Dialister* was most closely associated with RA. The *Bacteroidaceae* and *Marinifilaceae* families in phylum *Bacteroidota* were expanded in the HC group; and *Sutterella* and *Escherichia-Shigella* in phylum *Proteobacteria*, which formed separate clusters, were expanded in the RA and HC groups, respectively (Figures 7A,B).

### Functional Analysis of Gut Microbes in Rheumatoid Arthritis

**FIGURE 8:**
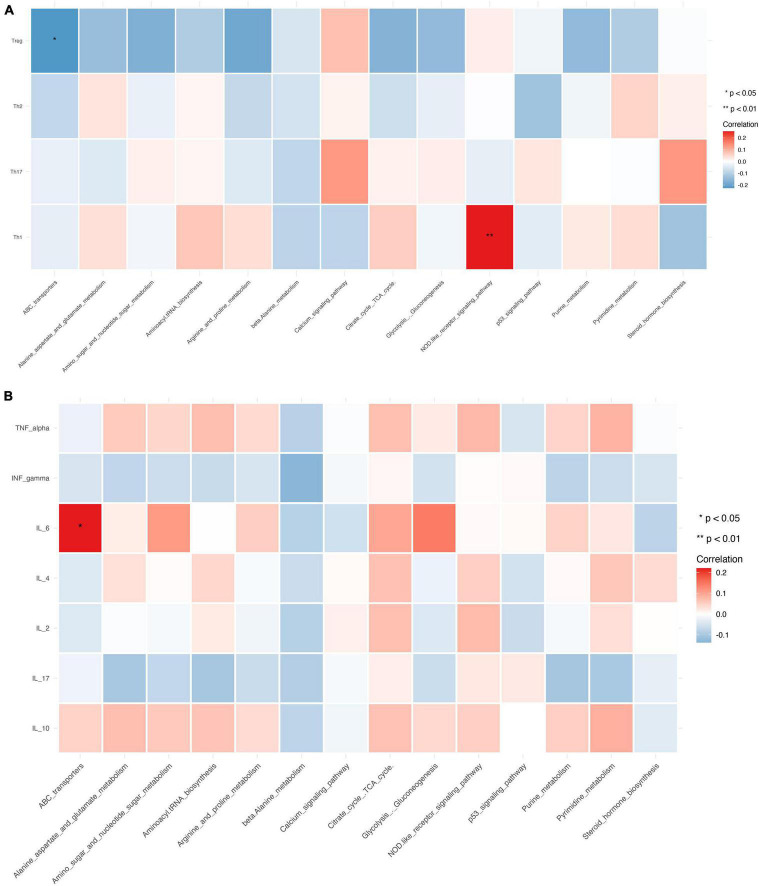
Relationship between CD4^+^ T cell subpopulations, cytokines, and metabolic pathways. **(A,B)** Heat map of the correlation between different metabolic pathways and CD4^+^ T cell subpopulations **(A)** and cytokines. Colors indicate the Spearman rank correlation, with lighter and darker colors corresponding to weaker and stronger correlations, respectively. *P < 0.05, **P < 0.01.

PICRUSt2 was used to infer the functions of the significantly different taxa between RA patients and HCs based on 16S rRNA gene sequences. KEGG pathways that were significantly enriched in RA patients included amino acid metabolism (e.g., alanine, aspartate, and glutamate), amino sugar and nucleotide sugar metabolism, β-alanine metabolism, ATP-binding cassette (ABC) transporters, glycolysis/gluconeogenesis, NOD-like receptor signaling pathway, and p53 signaling pathway (Figure 7C). The metabolic pathway of ABC transporters was negatively correlated with the absolute numbers of Tregs (*P* < 0.01), while IL-6 level and NOD-like receptor signaling pathway showed positive correlations with the absolute number of Th1 cells (*P* < 0.05) (Figures 8A,B).

### Relationship Between Gut Microbiota and CD4^+^ T Cell Subpopulations and Cytokine Levels

**FIGURE 9:**
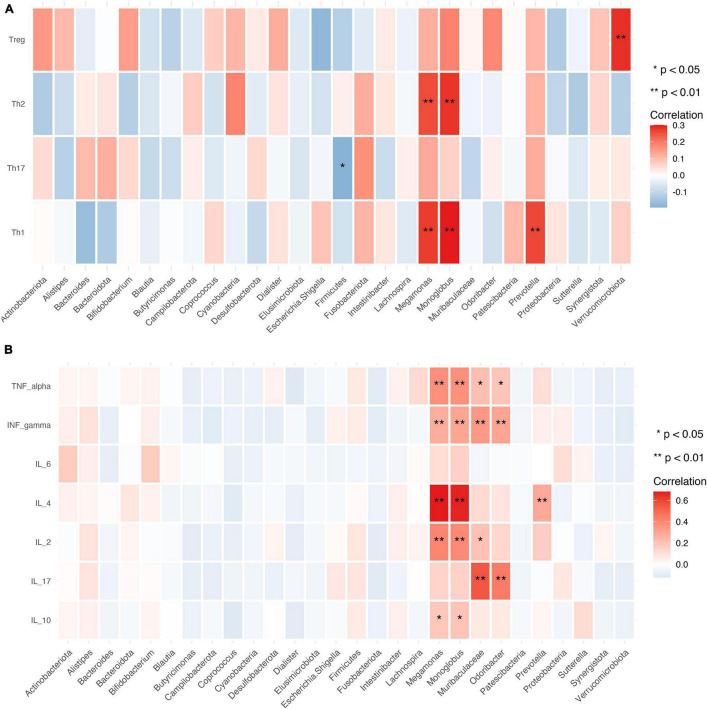
Relationship between gut microbiota, CD4^+^ T cell subpopulations, and cytokines. **(A,B)** Heat map of the correlation between different gut microbial species and CD4^+^ T cell subpopulations **(A)** and cytokines **(B)**. Colors indicate the Spearman rank correlation, with lighter and darker colors corresponding to weaker and stronger correlations, respectively. *P < 0.05, **P < 0.01.

The relative abundance of *Megamonas* and *Monoglobus* was positively correlated with the absolute numbers of Th1 and Th2 cells, which are CD4^+^ T cell subsets (*P* < 0.01). *Verrucomicrobiota* was positively correlated with the absolute number of Tregs (*P* < 0.01), while *Firmicutes* was negatively correlated with the absolute number of Th17 cells (*P* < 0.05) (Figure 9A).

Gut microbiota abundance was closely associated with cytokine levels. The relative abundance of *Megamonas* and *Monoglobus* showed significant positive correlations with the levels of IL-10, IL-2, IL-4, TNF-α, and IFN-γ (*P* < 0.01 or < 0.05). *Muribaculaceae* and *Odoribacter* were positively correlated with IL-17, and TNF-α, and IFN-γ levels (*P* < 0.01 or < 0.05) and *Prevotella* was positively correlated with IL-4 level (Figure 9B).

### Disease Activity in Rheumatoid Arthritis Is Closely Linked to Gut Microbiota, Lymphocyte Subpopulations, and Cytokine Levels

**FIGURE 10:**
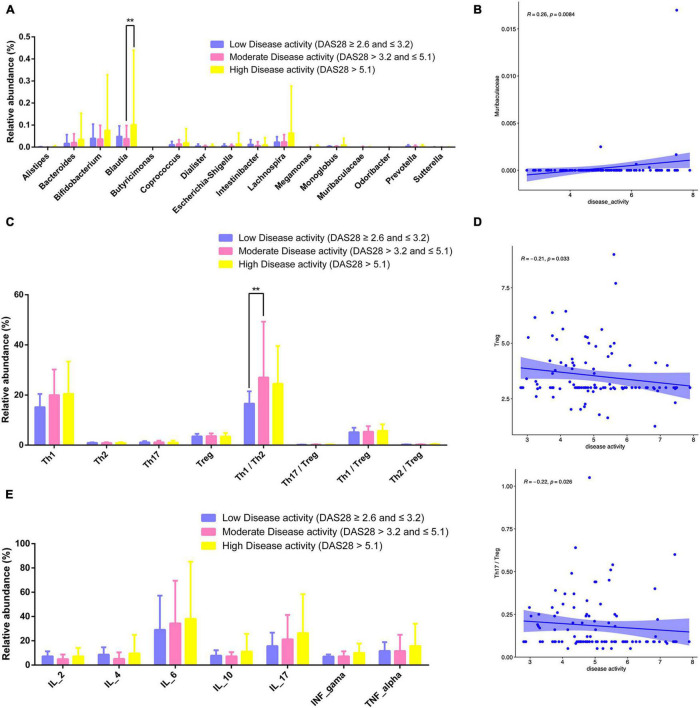
Gut microbiota, CD4^+^ T cell subpopulations, and cytokines in different RA disease activity groups. **(A)** Relative abundance of gut microbiota for different disease activity levels. **(B)** Scatter plot of the correlation between disease activity and Muribaculaceae. **(C)** Relative abundance of lymphocyte subpopulations for different disease activity levels. **(D)** Scatterplot of the correlation between disease activity and Treg counts Th17/Treg ratio. **(E)** Relative abundance of cytokines for different disease activity levels. **P < 0.01.

RA patients were classified according to disease activity into low (DAS28 ≥ 2.6 and ≤ 3.2), moderate (DAS28 > 3.2 and ≤ 5.1), and high (DAS28 > 5.1) activity groups. The relative abundance of *Bifidobacterium* and *Lachnospira* increased with disease activity (Figure 10A). The relative abundance of Blautia was higher in patients with high disease activity compared to those with moderate and low disease activity (*P* < 0.01; Figure 10A). *Muribaculaceae* abundance was positively correlated with disease activity (*P* = 0.0084; Figure 10B).

The number of Tregs and Th17/Tregs was inversely correlated with disease activity (*P* = 0.033 and 0.026; Figure 10D). The relative abundance of Th1/Th2 cells was higher patients with moderate disease activity than in those with low or high disease activity (*P* < 0.01; Figure 10C). Relative abundance of cytokines for different disease activity levels has been shown in Figure 10E.

## Discussion

The results of this study demonstrate that gut flora composition and α and β diversity were altered in RA patients, which is consistent with previous studies (Maeda and Takeda, 2019; Sun et al., 2019). RA patients significantly decreased α-diversity in the gut microbiome (Observed, Chao1, ACE, and Shannon), indicating that the richness and evenness of bacteria in RA groups were lower. Perturbation in the community was also found via the analysis of β-diversity (PCoA). As to beta diversity, PCoA results indicated that the composition of gut microbiota was significantly different in these two groups. At the phylum level, the profile of gut microbiota appeared dominated by *Firmicutes* and *Bacteroidetes* and the abundance of *Firmicutes*, *Fusobacteriota*, and *Bacteroidota* was shown to be decreased in RA patients (Kasselman et al., 2018) whereas that of *Actinobacteria* was higher (Chen et al., 2016). *Firmicutes*, a butyrate-producing bacterium, regulates the differentiation of Tregs that inhibit inflammation; thus, a reduction in *Firmicutes* abundance and consequent decrease in butyrate levels can lead to inflammation in RA patients. *Actinobacteria* was reported increased in the rheumatoid arthritis, some of which were consistent with our findings (Chen et al., 2016). Actinobacteria can predict the RA status (Chen et al., 2016), However, another study is in contrast to our findings (Jeong et al., 2019). The role of *Actinobacteria* expression and function in RA needed to investigate further.

At the genus level, the profile of gut microbiota appeared dominated by the abundance of *Faecalibacterium*, *Agathobacter*, *Roseburia*, *Subdoligranulum*, and *Bacteroidetes*. The relactive ahundnce of *Faecalibacterium* (phylum *Firmicutes*) showed reduced abundance in RA patients compared to HCs. *Faecalibacterium* produces butyrate, which maintains the integrity of the intestinal epithelial cell layer (Khan et al., 2012). *Collinsella* was also increased in RA patients. A recent study found that decreased *Faecalibacterium* and increased *Collinsella* abundance may enhance epithelial permeability, resulting in the entry of microbial products into the lamina propria and subepithelial space (Chen et al., 2016). Changes in the composition of gut microbiota are tightly linked with host immune status and local inflammation (Faber and Bäumler, 2014; Lee and Ko, 2016; Katayama et al., 2019). *Bacteroides*, which was decreased in RA patients compared to HCs, has been implicated in chronic inflammation and may cause osteomyelitis in RA (Sun et al., 2019). On the other hand, *Blautia* and *Lachnoclostridium* were increased in RA patients; *Lachnoclostridium* may contribute to the progression of inflammatory arthritis. *Dialister* has been linked to antidepressants drug along with *Coprococcus* and butyrate-producing *Faecalibacterium*. Whether there is reciprocal interaction in preventing autoimmune diseases requires further study. Above all, a correlation may exist between the abundance of these bacteria and RA patients.

The functions of gut microbiota showing altered abundance in RA patients included ABC transporters, glycolysis/gluconeogenesis, and NOD-like receptor signaling. ABC transporters enable the passage of endogenous and xenobiotic compounds through the cell membrane, which may be related to the development of rheumatic disease resistance and disease activity (Atisha-Fregoso et al., 2016). ABC transporters were shown to induce C-X-C chemokine receptor (CXCR) 4 overexpression in B cells and the production of various inflammatory cytokines such as TNF and IL-6 (Tsujimura et al., 2018). Glycolysis/gluconeogenesis plays an important role in maintaining adequate sugar sources (Cheng et al., 2017). The activation of NOD-like receptor signaling modulates the innate immune response and inflammation (Franca et al., 2016; Cheng et al., 2017; Root-Bernstein, 2020). On the other hand, steroid hormone biosynthesis (Cutolo et al., 2004) and p53 signaling were decreased in RA. Steroid hormone-related gene polymorphisms have been linked to bone erosion in rheumatoid arthritis (Sánchez-Maldonado et al., 2019). The p53 signaling pathway mediates the cellular response to stress, cell cycle, DNA repair, senescence, and apoptosis (Hou et al., 2016; Zhang et al., 2020). Bacteroides abundance was reduced in RA patients compared to HCs, suggesting that Bacteroides contributes to disease pathogenesis through the release of bioactive molecules such as butanoate, dicarboxylate, and glyoxylate (Wang et al., 2021). Notably, there was a close correlation between metabolic pathways and some genera (*Blautia*, *Escherichia-Shigella*, *Proteobacteria*, *Coprococcus*, and *Verrucomicrobiota*).

Intestinal microbiota are closely related to the immune profile of RA (Hu et al., 2017; Zhang et al., 2018; Li H. et al., 2021). *Lactobacillus casei* has been shown to alleviate arthritis by altering Treg/Th17 balance and modulating gut microbiota abundance and the plasma metabolome (Jhun et al., 2020; Fan et al., 2021). *Verrucomicrobia* (*Akkermansia muciniphila*) plays an anti-inflammatory role by regulating Treg differentiation and enhancing the production of short-chain fatty acids (Zhai et al., 2019). The study about *Verrucomicrobia* in RA is scarce, our finding may provide a new clue for the function of *Verrucomicrobia* in disease causation. It was reported that *Firmicutes-rich* suppresses inflammation and Th17 pathways (Natividad et al., 2015), suggesting that it has therapeutic potential in ulcerative colitis. *Bacteroides fragilis* and *Faecalibacterium prausnitzii* induce the secretion of IL-10 by CD4^+^ T cells (Mazmanian et al., 2005; Sokol et al., 2008). Gut dysbiosis caused by segmented filamentous bacteria may reduce the number of anti-inflammatory Tregs and increase the risk of autoimmunity (Scher et al., 2013). IL-10 is an anti-inflammatory cytokine secreted by various lymphocytes including Th1, Th2, and Th17 cells and Tregs (Mollazadeh et al., 2019). The dysregulation of IL-10 has been reported in various inflammatory diseases such as lupus, arthritis, and psoriasis. In our study, IL-10 level and Th1 and Th2 counts were positively correlated with *Megamonas* and *Monoglobus* abundance; meanwhile, the relative abundance of *Prevotella* and *Monoglobus* was positively correlated with the absolute number of Th1 and Th2 cells and Il-4, IL-2, IL-10, TNF-α, and IFN-γ levels. Improving the balance of gut microbiota with *Blautia* along with Bifidobacterium and Ruminococcus was shown to alleviate inflammation by suppressing Tregs (Picchianti-Diamanti et al., 2018). Our results speculate that gut microorganisms influence RA by modulating the immune system. This requires further studying.

We found that the abundance of *Escherichia-Shigella*, *Monoglobus*, and *Lachnospira* increased while that of *Intestinibacter* and *Prevotella* decreased with disease activity in RA. An increase in RA disease activity was shown to be associated with elevated levels of TNF-α and IFN-γ, a higher Th17 count, and altered Treg balance (i.e., Th17/Treg and Th2/Treg ratios) (Edavalath et al., 2016). IL-6, IL-17, and IL-10 levels were higher in RA patients than in HCs and IL-17 and IL-10 were positively correlated with DAS28 score, suggesting that these cytokines can serve as markers for disease outcome and inflammatory response (Ge et al., 2015; Marwa et al., 2017; Dhaouadi et al., 2018). In our study, IL-6 level was significantly higher in RA patients with a high DAS28 score compared to those with lower scores. Meanwhile, high IL-6 level was shown to be an independent risk factor for high disease activity in RA, as reflected by the DAS28 score (Park et al., 2016), and alters the Treg/Th17 balance by downregulating IL-6 (Samson et al., 2012). These results suggest that regulating cytokine levels and lymphocyte subpopulations may be an effective strategy in controlling disease activity in RA.

## Conclusion

The results of our study demonstrate that gut microbiota play an important role in the pathogenesis of RA, as evidenced by the observed correlations between gut microbiota and CD4^+^ T cell counts, cytokine levels, and disease activity in RA patients compared to HCs. We found that gut microbiota composition was altered in RA, which was primarily associated with changes in metabolic signaling that could contribute to imbalances in CD4^+^ T cell subpopulations and cytokines and affect disease activity. These findings provide novel insight into the possible pathogenic mechanisms of RA and suggest that the disease can potentially be regulated by affecting CD4^+^ T cell subpopulations and cytokines through regulation of the gut microbiome profile.

## Data Availability Statement

The original contributions presented in the study are included in the article/supplementary material, further inquiries can be directed to the corresponding author/s.

## Ethics Statement

The studies involving human participants were reviewed and approved by The Second Affiliated Hospital of Shanxi Medical University. The patients/participants provided their written informed consent to participate in this study.

## Author Contributions

QW designed the study and wrote the manuscript. S-XZ, M-JC, and JQ performed the experiments. QW and JQ analyzed the data. C-HW, X-FL, QY, and P-FH contributed to manuscript revision, read, and approved the submitted version. All authors contributed to the article and approved the submitted version.

## Conflict of Interest

The authors declare that the research was conducted in the absence of any commercial or financial relationships that could be construed as a potential conflict of interest.

## Publisher’s Note

All claims expressed in this article are solely those of the authors and do not necessarily represent those of their affiliated organizations, or those of the publisher, the editors and the reviewers. Any product that may be evaluated in this article, or claim that may be made by its manufacturer, is not guaranteed or endorsed by the publisher.

## References

[B1] Abdulla O. A. Neamah W. Sultan M. Alghetaa H. K. Singh N. Busbee P. B. (2021). The Ability of AhR Ligands to Attenuate Delayed Type Hypersensitivity Reaction Is Associated With Alterations in the Gut Microbiota. *Front. Immunol.* 12:684727. 10.3389/fimmu.2021.684727 34267755 PMC8277436

[B2] Ahmad A. Yang W. Chen G. Shafiq M. Javed S. Ali Zaidi S. S. (2019). Analysis of gut microbiota of obese individuals with type 2 diabetes and healthy individuals. *PLoS One* 14:e0226372. 10.1371/journal.pone.0226372 31891582 PMC6938335

[B3] Anuradha R. Munisankar S. Bhootra Y. Dolla C. Kumaran P. Nutman T. B. (2017). Modulation of Mycobacterium tuberculosis-specific humoral immune responses is associated with Strongyloides stercoralis co-infection. *PLoS Negl. Trop. Dis.* 11:e0005569. 10.1371/journal.pntd.0005569 28459817 PMC5426788

[B4] Atisha-Fregoso Y. Lima G. Pascual-Ramos V. Baños-Peláez M. Fragoso-Loyo H. Jakez-Ocampo J. (2016). Rheumatoid Arthritis Disease Activity Is Determinant for ABCB1 and ABCG2 Drug-Efflux Transporters Function. *PLoS One* 11:e0159556. 10.1371/journal.pone.0159556 27442114 PMC4956301

[B5] Chen J. Li J. Gao H. Wang C. Luo J. Lv Z. (2012). Comprehensive evaluation of different T-helper cell subsets differentiation and function in rheumatoid arthritis. *J. Biomed. Biotechnol.* 2012:535361. 10.1155/2012/535361 23091349 PMC3469210

[B6] Chen J. Wright K. Davis J. M. Jeraldo P. Marietta E. V. Murray J. (2016). An expansion of rare lineage intestinal microbes characterizes rheumatoid arthritis. *Genome Med.* 8:43. 10.1186/s13073-016-0299-7 27102666 PMC4840970

[B7] Chen Z. Wong P. Y. Ng C. W. K. Lan L. Fung S. Li J. W. (2020). The Intersection between Oral Microbiota, Host Gene Methylation and Patient Outcomes in Head and Neck Squamous Cell Carcinoma. *Cancers* 12:3425. 10.3390/cancers12113425 33218162 PMC7698865

[B8] Cheng B. Zheng H. Wu F. Wu J. Liu X. Tang C. (2017). Metabolomics analysis of Danggui Sini decoction on treatment of collagen-induced arthritis in rats. *J. Chromatogr. B Analyt. Technol. Biomed. Life Sci.* 1061-1062 282–291. 10.1016/j.jchromb.2017.07.043 28763759

[B9] Chu H. Mazmanian S. K. (2013). Innate immune recognition of the microbiota promotes host-microbial symbiosis. *Nat. Immunol.* 14 668–675. 10.1038/ni.2635 23778794 PMC4109969

[B10] Cutolo M. Villaggio B. Seriolo B. Montagna P. Capellino S. Straub R. H. (2004). Synovial fluid estrogens in rheumatoid arthritis. *Autoimmun. Rev.* 3 193–198. 10.1016/j.autrev.2003.08.003 15110231

[B11] Dhaouadi T. Chahbi M. Haouami Y. Sfar I. Abdelmoula L. Ben Abdallah T. (2018). IL-17A, IL-17RC polymorphisms and IL17 plasma levels in Tunisian patients with rheumatoid arthritis. *PLoS One* 13:e0194883. 10.1371/journal.pone.0194883 29584788 PMC5870983

[B12] Douglas G. M. Maffei V. J. Zaneveld J. R. Yurgel S. N. Brown J. R. Taylor C. M. (2020). PICRUSt2 for prediction of metagenome functions. *Nat. Biotechnol.* 38 685–688. 10.1038/s41587-020-0548-6 32483366 PMC7365738

[B13] Edavalath S. Singh A. Soni N. Mohindra N. Kumar S. Misra R. (2016). Peripheral blood T helper type 17 frequency shows an inverse correlation with disease activity and magnetic resonance imaging-based osteitis and erosions in disease-modifying anti-rheumatic drug- and steroid-naive established rheumatoid arthritis. *Clin. Exp. Immunol.* 186 313–320. 10.1111/cei.12860 27568583 PMC5108076

[B14] England B. R. Thiele G. M. Anderson D. R. Mikuls T. R. (2018). Increased cardiovascular risk in rheumatoid arthritis: mechanisms and implications. *BMJ* 361:k1036. 10.1136/bmj.k1036 29685876 PMC6889899

[B15] Faber F. Bäumler A. J. (2014). The impact of intestinal inflammation on the nutritional environment of the gut microbiota. *Immunol. Lett.* 162 48–53. 10.1016/j.imlet.2014.04.014 24803011 PMC4219934

[B16] Fan Z. Ross R. P. Stanton C. Hou B. Zhao J. Zhang H. (2021). Lactobacillus casei CCFM1074 Alleviates Collagen-Induced Arthritis in Rats via Balancing Treg/Th17 and Modulating the Metabolites and Gut Microbiota. *Front. Immunol.* 12:680073. 10.3389/fimmu.2021.680073 34079556 PMC8165437

[B17] Franca R. Vieira S. M. Talbot J. Peres R. S. Pinto L. G. Zamboni D. S. (2016). Expression and activity of NOD1 and NOD2/RIPK2 signalling in mononuclear cells from patients with rheumatoid arthritis. *Scand. J. Rheumatol.* 45 8–12. 10.3109/03009742.2015.1047403 26202066

[B18] Frisbee A. L. Saleh M. M. Young M. K. Leslie J. L. Simpson M. E. Abhyankar M. M. (2019). IL-33 drives group 2 innate lymphoid cell-mediated protection during Clostridium difficile infection. *Nat. Commun.* 10:2712. 10.1038/s41467-019-10733-9 31221971 PMC6586630

[B19] Gabriel B. Medin C. Alves J. Nduati R. Bosire R. K. Wamalwa D. (2019). Analysis of the TCR Repertoire in HIV-Exposed but Uninfected Infants. *Sci. Rep.* 9:11954. 10.1038/s41598-019-48434-4 31420576 PMC6697688

[B20] Ge L. Huang Y. Zhang H. Liu R. Xu N. (2015). Association between polymorphisms of interleukin 10 with inflammatory biomarkers in East Chinese Han patients with rheumatoid arthritis. *Joint Bone Spine* 82 182–186. 10.1016/j.jbspin.2014.11.007 25623518

[B21] Hou J. Ouyang Y. Deng H. Chen Z. Song B. Xie Z. (2016). Whole-Genome Expression Analysis and Signal Pathway Screening of Synovium-Derived Mesenchymal Stromal Cells in Rheumatoid Arthritis. *Stem Cells Int.* 2016:1375031. 10.1155/2016/1375031 27642302 PMC5014955

[B22] Hu L. Geng S. Li Y. Cheng S. Fu X. Yue X. (2017). Exogenous Fecal Microbiota Transplantation from Local Adult Pigs to Crossbred Newborn Piglets. *Front. Microbiol.* 8:2663. 10.3389/fmicb.2017.02663 29375527 PMC5767267

[B23] Jeong Y. Kim J. W. You H. J. Park S. J. Lee J. Ju J. H. (2019). Gut Microbial Composition and Function Are Altered in Patients with Early Rheumatoid Arthritis. *J. Clin. Med.* 8:693. 10.3390/jcm8050693 31100891 PMC6572219

[B24] Jhun J. Min H. K. Ryu J. Lee S. Y. Ryu J. G. Choi J. W. (2020). Lactobacillus sakei suppresses collagen-induced arthritis and modulates the differentiation of T helper 17 cells and regulatory B cells. *J. Transl. Med.* 18:317. 10.1186/s12967-020-02477-8 32799896 PMC7429687

[B25] Kasselman L. J. Vernice N. A. DeLeon J. Reiss A. B. (2018). The gut microbiome and elevated cardiovascular risk in obesity and autoimmunity. *Atherosclerosis* 271 203–213. 10.1016/j.atherosclerosis.2018.02.036 29524863

[B26] Katayama Y. Yamada T. Tanimura K. Yoshimura A. Takeda T. Chihara Y. (2019). Impact of bowel movement condition on immune checkpoint inhibitor efficacy in patients with advanced non-small cell lung cancer. *Thorac. Cancer* 10 526–532. 10.1111/1759-7714.12969 30666802 PMC6397896

[B27] Khan M. T. Duncan S. H. Stams A. J. van Dijl J. M. Flint H. J. Harmsen H. J. (2012). The gut anaerobe *Faecalibacterium prausnitzii* uses an extracellular electron shuttle to grow at oxic-anoxic interphases. *ISME J.* 6 1578–1585. 10.1038/ismej.2012.5 22357539 PMC3400418

[B28] Kojima A. Tanaka-Kojima Y. Sakakura T. Nishizuka Y. (1976). Prevention of postthymectomy autoimmune thyroiditis in mice. *Lab. Invest.* 34 601–605. 778485

[B29] Lee H. Ko G. (2016). Antiviral effect of vitamin A on norovirus infection via modulation of the gut microbiome. *Sci. Rep.* 6:25835. 10.1038/srep25835 27180604 PMC4867650

[B30] Lee N. Kim W. U. (2017). Microbiota in T-cell homeostasis and inflammatory diseases. *Exp. Mol. Med.* 49:e340. 10.1038/emm.2017.36 28546563 PMC5454441

[B31] Li B. Guo Q. Wang Y. Su R. Gao C. Zhao J. (2020). Increased Serum Interleukin-2 Levels Are Associated with Abnormal Peripheral Blood Natural Killer Cell Levels in Patients with Active Rheumatoid Arthritis. *Mediators Inflamm.* 2020:6108342. 10.1155/2020/6108342 33013198 PMC7512106

[B32] Li H. Li S. Fan S. Xu Y. Tian X. (2021). Profiling intestinal microbiota of Metaplax longipes and Helice japonica and their co-occurrence relationships with habitat microbes. *Sci. Rep.* 11:21217. 10.1038/s41598-021-00810-9 34707208 PMC8551266

[B33] Li Y. Zhang S. X. Yin X. F. Zhang M. X. Qiao J. Xin X. H. (2021). The Gut Microbiota and Its Relevance to Peripheral Lymphocyte Subpopulations and Cytokines in Patients with Rheumatoid Arthritis. *J. Immunol. Res.* 2021:6665563. 10.1155/2021/6665563 33506059 PMC7810541

[B34] Li M. Wang F. (2021). Role of Intestinal Microbiota on Gut Homeostasis and Rheumatoid Arthritis. *J. Immunol. Res.* 2021:8167283. 10.1155/2021/8167283 34195296 PMC8203374

[B35] Li S. Wang Z. Yang Y. Yang S. Yao C. Liu K. (2017). Lachnospiraceae shift in the microbial community of mice faecal sample effects on water immersion restraint stress. *AMB Express* 7:82. 10.1186/s13568-017-0383-4 28417435 PMC5393979

[B36] Liang C. Tseng H. C. Chen H. M. Wang W. C. Chiu C. M. Chang J. Y. (2017). Diversity and enterotype in gut bacterial community of adults in Taiwan. *BMC Genomics* 18:932. 10.1186/s12864-016-3261-6 28198673 PMC5310273

[B37] Maeda Y. Takeda K. (2019). Host-microbiota interactions in rheumatoid arthritis. *Exp. Mol. Med.* 51 1–6. 10.1038/s12276-019-0283-6 31827063 PMC6906371

[B38] Marwa O. S. Kalthoum T. Wajih K. Kamel H. (2017). Association of IL17A and IL17F genes with rheumatoid arthritis disease and the impact of genetic polymorphisms on response to treatment. *Immunol. Lett.* 183 24–36. 10.1016/j.imlet.2017.01.013 28143790

[B39] Mazmanian S. K. Liu C. H. Tzianabos A. O. Kasper D. L. (2005). An immunomodulatory molecule of symbiotic bacteria directs maturation of the host immune system. *Cell* 122 107–118. 10.1016/j.cell.2005.05.007 16009137

[B40] Mollazadeh H. Cicero A. F. G. Blesso C. N. Pirro M. Majeed M. Sahebkar A. (2019). Immune modulation by curcumin: the role of interleukin-10. *Crit. Rev. Food Sci. Nutr.* 59 89–101. 10.1080/10408398.2017.1358139 28799796

[B41] Muraoka W. T. Granados J. C. Gomez B. I. Nicholson S. E. Chung K. K. Shupp J. W. (2020). Burn resuscitation strategy influences the gut microbiota-liver axis in swine. *Sci. Rep.* 10:15655. 10.1038/s41598-020-72511-8 32973266 PMC7515893

[B42] Natividad J. M. Pinto-Sanchez M. I. Galipeau H. J. Jury J. Jordana M. Reinisch W. (2015). Ecobiotherapy Rich in Firmicutes Decreases Susceptibility to Colitis in a Humanized Gnotobiotic Mouse Model. *Inflamm. Bowel Dis.* 21 1883–1893. 10.1097/mib.0000000000000422 26060932

[B43] Niu H. Q. Yuan C. Yan C. Li N. Lei Y. S. Li X. (2021). Decreased numbers and sex-based differences of circulating regulatory T cells in patients with seropositive undifferentiated arthritis. *Ther. Adv. Chronic Dis.* 12:2040622320986721. 10.1177/2040622320986721 33717426 PMC7925950

[B44] Niu H. Q. Zhao X. C. Li W. Xie J. F. Liu X. Q. Luo J. (2020). Characteristics and reference ranges of CD4(+)T cell subpopulations among healthy adult Han Chinese in Shanxi Province, North China. *BMC Immunol.* 21:44. 10.1186/s12865-020-00374-9 32746780 PMC7397677

[B45] Ott B. M. Rickards A. Gehrke L. Rio R. V. (2014). Characterization of shed medicinal leech mucus reveals a diverse microbiota. *Front. Microbiol.* 5:757. 10.3389/fmicb.2014.00757 25620963 PMC4288373

[B46] Park Y. J. Yoo S. A. Kim G. R. Cho C. S. Kim W. U. (2016). Urinary interleukin-6 as a predictor of radiographic progression in rheumatoid arthritis: A 3-year evaluation. *Sci. Rep.* 6:35242. 10.1038/srep35242 27731382 PMC5059680

[B47] Picchianti-Diamanti A. Panebianco C. Salemi S. Sorgi M. L. Di Rosa R. Tropea A. (2018). Analysis of Gut Microbiota in Rheumatoid Arthritis Patients: disease-Related Dysbiosis and Modifications Induced by Etanercept. *Int. J. Mol. Sci.* 19:2938. 10.3390/ijms19102938 30261687 PMC6213034

[B48] Ramadan M. Hetta H. F. Saleh M. M. Ali M. E. Ahmed A. A. Salah M. (2021). Alterations in skin microbiome mediated by radiotherapy and their potential roles in the prognosis of radiotherapy-induced dermatitis: a pilot study. *Sci. Rep.* 11:5179. 10.1038/s41598-021-84529-7 33664352 PMC7933139

[B49] Root-Bernstein R. (2020). Synergistic Activation of Toll-Like and NOD Receptors by Complementary Antigens as Facilitators of Autoimmune Disease: review, Model and Novel Predictions. *Int. J. Mol. Sci.* 21:4645. 10.3390/ijms21134645 32629865 PMC7369971

[B50] Samson M. Audia S. Janikashvili N. Ciudad M. Trad M. Fraszczak J. (2012). Brief report: inhibition of interleukin-6 function corrects Th17/Treg cell imbalance in patients with rheumatoid arthritis. *Arthritis Rheum.* 64 2499–2503. 10.1002/art.34477 22488116

[B51] Sánchez-Maldonado J. M. Cáliz R. Canet L. Horst R. T. Bakker O. den Broeder A. A. (2019). Steroid hormone-related polymorphisms associate with the development of bone erosions in rheumatoid arthritis and help to predict disease progression: results from the REPAIR consortium. *Sci. Rep.* 9:14812. 10.1038/s41598-019-51255-0 31616008 PMC6794376

[B52] Scher J. U. Sczesnak A. Longman R. S. Segata N. Ubeda C. Bielski C. (2013). Expansion of intestinal Prevotella copri correlates with enhanced susceptibility to arthritis. *Elife* 2:e01202. 10.7554/eLife.01202 24192039 PMC3816614

[B53] Sokol H. Pigneur B. Watterlot L. Lakhdari O. Bermúdez-Humarán L. G. Gratadoux J. J. (2008). *Faecalibacterium prausnitzii* is an anti-inflammatory commensal bacterium identified by gut microbiota analysis of Crohn disease patients. *Proc. Natl. Acad. Sci. U. S. A.* 105 16731–16736. 10.1073/pnas.0804812105 18936492 PMC2575488

[B54] Suárez-Moo P. Cruz-Rosales M. Ibarra-Laclette E. Desgarennes D. Huerta C. Lamelas A. (2020). Diversity and Composition of the Gut Microbiota in the Developmental Stages of the Dung Beetle Copris incertus Say (*Coleoptera*, *Scarabaeidae*). *Front. Microbiol.* 11:1698. 10.3389/fmicb.2020.01698 32793162 PMC7393143

[B55] Sun Y. Chen Q. Lin P. Xu R. He D. Ji W. (2019). Characteristics of Gut Microbiota in Patients With Rheumatoid Arthritis in Shanghai, China. *Front. Cell. Infect. Microbiol.* 9:369. 10.3389/fcimb.2019.00369 31709198 PMC6819506

[B56] Taneja V. (2014). Arthritis susceptibility and the gut microbiome. *FEBS Lett.* 588 4244–4249. 10.1016/j.febslet.2014.05.034 24873878 PMC4246018

[B57] Tsujimura S. Adachi T. Saito K. Kawabe A. Tanaka Y. (2018). Relevance of P-glycoprotein on CXCR4(+) B cells to organ manifestation in highly active rheumatoid arthritis. *Mod. Rheumatol.* 28 276–286. 10.1080/14397595.2017.1341458 28696805

[B58] Vaahtovuo J. Munukka E. Korkeamäki M. Luukkainen R. Toivanen P. (2008). Fecal microbiota in early rheumatoid arthritis. *J. Rheumatol.* 35 1500–1505. 18528968

[B59] Wang H. Ong E. Kao J. Y. Sun D. He Y. (2021). Reverse Microbiomics: a New Reverse Dysbiosis Analysis Strategy and Its Usage in Prediction of Autoantigens and Virulent Factors in Dysbiotic Gut Microbiomes From Rheumatoid Arthritis Patients. *Front. Microbiol.* 12:633732. 10.3389/fmicb.2021.633732 33717026 PMC7947680

[B60] Zhai R. Xue X. Zhang L. Yang X. Zhao L. Zhang C. (2019). Strain-Specific Anti-inflammatory Properties of Two Akkermansia muciniphila Strains on Chronic Colitis in Mice. *Front. Cell. Infect. Microbiol.* 9:239. 10.3389/fcimb.2019.00239 31334133 PMC6624636

[B61] Zhang K. X. Ip C. K. Chung S. K. Lei K. K. Zhang Y. Q. Liu L. (2020). Drug-resistance in rheumatoid arthritis: the role of p53 gene mutations, ABC family transporters and personal factors. *Curr. Opin. Pharmacol.* 54 59–71. 10.1016/j.coph.2020.08.002 32942096

[B62] Zhang Q. Li C. Niu X. Zhang Z. Li F. Li F. (2018). An intensive milk replacer feeding program benefits immune response and intestinal microbiota of lambs during weaning. *BMC Vet. Res.* 14:366. 10.1186/s12917-018-1691-x 30477479 PMC6258415

[B63] Zhang S. X. Wang J. Wang C. H. Jia R. H. Yan M. Hu F. Y. (2021). Low-dose IL-2 therapy limits the reduction in absolute numbers of circulating regulatory T cells in rheumatoid arthritis. *Ther. Adv. Musculoskelet. Dis.* 13:1759720x211011370. 10.1177/1759720x211011370 33995604 PMC8107675

[B64] Zhao C. Chu Y. Liang Z. Zhang B. Wang X. Jing X. (2019). Low dose of IL-2 combined with rapamycin restores and maintains the long-term balance of Th17/Treg cells in refractory SLE patients. *BMC Immunol.* 20:32. 10.1186/s12865-019-0305-0 31484501 PMC6727508

